# Psychosomatic medicine and the philosophy of life

**DOI:** 10.1186/1747-5341-5-2

**Published:** 2010-01-21

**Authors:** Michael A Schwartz, Osborne P Wiggins

**Affiliations:** 1 Department of Psychiatry, University of Hawaii 1106 Blackacre Trail, Austin, Texas, 78746, USA; 2 Department of Philosophy, University of Louisville, Louisville, Kentucky, 40292, USA

## Abstract

Basing ourselves on the writings of Hans Jonas, we offer to psychosomatic medicine a philosophy of life that surmounts the mind-body dualism which has plagued Western thought since the origins of modern science in seventeenth century Europe. Any present-day account of reality must draw upon everything we know about the living and the non-living. Since we are living beings ourselves, we know what it means to be alive from our own first-hand experience. Therefore, our philosophy of life, in addition to starting with what empirical science tells us about inorganic and organic reality, must also begin from our own direct experience of life in ourselves and in others; it can then show how the two meet in the living being. Since life is ultimately one reality, our theory must reintegrate psyche with soma such that no component of the whole is short-changed, neither the objective nor the subjective. In this essay, we lay out the foundational components of such a theory by clarifying the defining features of living beings as *polarities*. We describe three such polarities:

1) Being vs. non-being: Always threatened by non-being, the organism must constantly re-assert its being through its own activity.

2) World-relatedness vs. self-enclosure: Living beings are both enclosed with themselves, defined by the boundaries that separate them from their environment, while they are also ceaselessly reaching out to their environment and engaging in transactions with it.

3) Dependence vs. independence: Living beings are both dependent on the material components that constitute them at any given moment and independent of any particular groupings of these components over time.

We then discuss important features of the polarities of life: Metabolism; organic structure; enclosure by a semi-permeable membrane; distinction between "self" and "other"; autonomy; neediness; teleology; sensitivity; values. Moral needs and values already arise at the most basic levels of life, even if only human beings can recognize such values as moral requirements and develop responses to them.

## The Evolution of a Method for Studying Life

Psychosomatic medicine requires a philosophy that surmounts mind-body dualism. Such a dualism has plagued Western thought since the origins of modern science in seventeenth century Europe. In its youth modern science perhaps needed to separate mind from body in order to separate religious concerns from scientific ones. If religion was concerned mainly with the human soul, then Descartes' claim that the human soul *(res cogitans) *differed *in toto *from physical reality *(res extensa) *implied that the science of physics had no relevance for the human soul. As long as science promised not to encroach on discussions of the human soul, it found itself free to investigate physical matter in an entirely new way. Hence modern science could abandon the Aristotelian conception of nature that the Catholic Church continued to advocate because science conceded to the Church a monopoly on all questions of the spirit. Metaphysical dualism thus won a tolerance for new modes of thought by parceling reality out to two entirely separate conceptual systems, one theological and the other scientific and mathematical [1-3].

This peaceful co-existence of theology and science was always threatened by deep strains, however, because Cartesian dualism was from the outset challenged by warring monisms. Repeated failures to explain the *relationship *between mind and body made dualism seem implausible because it was obvious from our own daily experience that mind and body were intimately united. But since dualism had insisted on an absolute difference between mind and body, philosophers and theologians felt themselves forced to take either one side or the other. Materialists would insist that physical nature alone had true being and that mind was mere appearance. Idealists would counter by deeming mind the sole reality and everything physical a mere idea thought by mind. In struggling thus to overcome dualism materialists and idealists simply presupposed the options dualism forced upon them. For each monism took it for granted that the only alternatives for defining reality were *either *matter *or *mind. Nothing else seemed conceivable [1].

Darwin's theory of evolution, however, undermined the peace that metaphysical dualism had sought to establish. Darwinian Theory explained the human soul as having evolved through the same regular processes of chance mutation and natural selection that had produced all other living beings. Hence the human soul was incorporated back into the animal kingdom, and as a consequence the soul required no other explanation than that which science could now offer. Empirical science thereby became *universal: all of reality *could be understood in the same basic scientific terms and laws [4,5].

This universalizing of scientific conceptualization seemed to betoken the victory of *metaphysical materialism*. If all of reality could be explained by science, then all of reality could ultimately be explained in terms of the most basic constituents that science had uncovered, namely, inorganic matter. Hence we need not speak of "mind," "spirit," or "soul" anymore except to demonstrate how even these subjective appearances could be accounted for fully by a law-governed physical causality.

But, as Hans Jonas has shown [4], there is another way to interpret the incorporation of the whole human being back into scientific theory. If, in this post-Darwinian age, we must now account for everything living and non-living in a unified system of thought, then we should be able to draw on everything we know about the living and non-living in our account of reality. This should include everything that we encounter of the living and non-living in our own direct experience. And here we can claim *privileged access: since we are living beings ourselves, we know what it means to be alive from our own first-hand experience*. Every moment of our waking lives we directly experience life, life in ourselves and in others. Our most intimate experience of life is in our own individual lives. But this constant experience of our own being-alive makes it possible for us to make sense of the being-alive of other people and, to some extent, of animals. Hence we should be able to start from both sides -- from the side of what empirical science can tell us about inorganic and organic reality and from the side of our own direct experience of life in ourselves and in others - and show how the two meet in the living being. If dualism has been undermined, then we must strive for a unified understanding of life, an understanding that fully appreciates both the biological processes of the organism and the inward, felt experiences of being-alive. Hence, aiming at their intersection, we shall reason from both directions. We do this in the confidence that life is ultimately *one *reality, however complex. Human beings are *psychosomatic wholes*, and therefore a theory that reintegrates *psyche *with *soma *can be developed as long as no component of the whole is short-changed. We shall search for features that characterize *life as such*, whether "objective" or "subjective." These features of living beings in general emerge, in our view, as *polarities*. Living beings exist as suspended between opposite poles of reality. We shall now attempt to describe some of these vital polarities [4].

## Polarities of Life

The existence of every living being is sustained through metabolism. Unlike inorganic matter, the very being of a living entity is contingent upon its own ceaseless activity [1]. As a result the existence of the organism from moment to moment is its own *dynamic achievement*. Inorganic matter need not *actively do *anything in order to endure as the being it is, but organisms must. This inescapable need to persistently bring about their own continuation through their own metabolic functioning proves that organisms are *threatened *beings: if they do not actively achieve and repeatedly re-achieve their own reality, they die. Ceaselessly dependent on their own functioning for their survival, organisms hang suspended over the abyss of non-being. Hence we can acknowledge one of the polarities that define life: *always threatened by non-being the organism must constantly re-assert its being through its own activity *[6-8].

This activity, however, must be an *organized *activity. Metabolic processes are *structured *processes, and it is this very structure and the processes it channels that must be maintained as such. When the structure fails to determine the direction of the processes, the organism dies. Accordingly, the identity of the organism depends on the maintenance of its internal structure. We might even say that the identity of the organism *is *the identity of the structure. This becomes more obvious when we note that the components that constitute the organism are constantly changing. The material components of the organism come and go, but it is important that the organism remain as the same one. To "remain as the same one" is to maintain the same structure even in the midst of constant change of components. In order to maintain this constant change of its components, however, the organism must to some extent be open to its environment, the ultimate source of the components. We are now in a position to appreciate one of the distinctive polarities of living beings. *Living beings are both enclosed within themselves, defined by the boundaries that separate them from their environment, while they are also ceaselessly reaching out to their environment and transacting with it *[7]. This polarity is found even in the single cell.

On the one hand, the cell membrane determines the cell's boundaries: the reality of the cell extends no farther than this membrane. And indeed these boundaries must be maintained if the cell is to continue to be. If the membrane breaks down sufficiently, the cell dies. Hence the membrane must maintain the separation of the cell from the rest of reality. Death consists in the loss of this separation. This need to remain bounded and distinct from that which is outside is observed at all levels of organic life. From the single cell, through the different organs of animal bodies, to the level of human beings as whole persons, "self" and "other" are definitely distinguished [6]. This distinction between self and other is demonstrated most clearly, of course, in the immune system. The immune system is geared to detect what is non-self; and once this detection of otherness occurs, the immune system actively opposes the invader.

On the other hand, the membrane is semi-permeable so that the cell may continually exchange its material with realities outside of it. Literally *through *its membrane the cell metabolically interacts with that which is not itself. Indeed this interaction with other entities is necessary if the cell is to maintain its existence: the cell is physically dependent upon the outside for its continuation in being. This dependency on what is not itself in order to survive evinces the organism's *neediness*: lacking self-sufficiency, the living being must of necessity acquire the means for its existence from its environment. However, this unavoidable exposure to the environment, born out of need, manifests again the riskiness of organic existence. The environment can prove harmful and even deadly. The alien and uncontrollable nature of the environment poses an additional threat to the already precarious venture that is organic life. Hence the cell is both enclosed within its own boundaries in order to maintain its separate and autonomous being while it must also interact with outside realities and indeed even exchange its own matter with them [6].

Through the metabolic exchange of material components the cell undergoes ceaseless change in its physicochemical make-up. But this change is, as we have seen, an *organized *change: it is determined by the *internal structure *of the cell. Through the change, then, the cell maintains its own separate identity while it also changes the physicochemical parts that compose it. It is both in flux and stable. Maintaining its stable identity through ceaseless turnover in its material constituents, the being of the organism is both independent of and dependent on these constituents. *Some *material constituents are always necessary for the existence of the organism; hence the *dependence *of the organism. But since these constituents will eventually be exchanged for others as the organism continues to live, the organism is *independent *of precisely *these *constituents, i.e., of whichever constituents compose it at any given time. We can therefore recognize one of the other polarities of living beings: *they are both dependent on the material components that constitute them at any given moment and independent of any particular groupings of these components across time *[6,7]. This polarity of dependence and independence always permeates organic existence.

As we have said, the metabolic activity of the organism is geared toward sustaining the existence of the organism. This being geared toward the sustaining of its own being shows that the metabolism of the organism is "for the sake of" its own continuation in being. The being that the activity is geared toward preserving is the organism's *future *being. The metabolic functioning is for the sake of bridging the temporal gap that separates the organism in the present from its own existence in the future. In slightly different terms, metabolic activity serves the *temporal enduring *of the organism. Hence it is temporal duration that poses the main threat to the organism's contingent existence: the question of whether the organism will endure from moment to moment always remains an unanswered question until the future becomes the present and the organism still lives. And the threat can be defeated only if the activity of metabolism is sustained. Life is thus *teleological*: the present activity of the living being *aims at *its own future being [8,9].

If we can speak of the metabolic activity of the organism as occurring "for the sake of" the organism's future being, this means that at some fundamental level the organism posits its own continuation in reality as a "good." In other words, the organism posits its own existence as having a positive value. Value is thus built into the reality of organic life: it is organic life itself that places value there. It is not human beings and certainly not human agency that introduces value into an otherwise value-free universe. Living beings themselves, by striving to preserve themselves, already signal that, at least for the being involved, its own life is a good [10-12].

We can see, then, that the values that motivate medical practice are grounded in organic life itself. While only human beings can develop and practice medical treatment, it is not human beings who introduce into the world the values that call for and justify that treatment. Living beings themselves posit the goodness of an activity that prevents death and alleviates suffering. If for the organism its own continuation is good, then its death would be bad. Hence the *moral *need to combat death issues from the organism's own internal striving. And therefore the need to treat and hopefully cure the ill organism so that it does not die - at least not before its naturally allotted time - is based on a value that the organism itself posits. The same would be true for suffering and pain, at least for those organism's that can *feel*. *Felt *suffering and pain are posited by the organism feeling them as bad. Hence the moral need to relieve and even eradicate pain through medical treatment arises at the most basic levels of life, even if only human beings can recognize this value as a moral requirement and develop the medical techniques to respond to it [11,13].

The historian of biology, Georges Canguilheim, also insists on this positive value of health and negative value of illness as posited by the organism itself and not simply through some external judgment conceived by medical practitioners [14]. Canguilheim finds this evaluation rendered by living beings themselves to underlie the fundamental distinction between the "normal" and the "pathological" states of the organism. Medicine simply draws on this basic distinction rendered by life itself on itself in developing scientific and technical means for treating them. Canguilheim writes,

We think that medicine exists as the art of life because the living human being himself calls certain dreaded states or behaviors pathological (hence requiring avoidance or correction) relative to the dynamic polarity of life, in the form of a negative value. We think that in doing this the living human being, in a more or less lucid way, extends a spontaneous effort, peculiar to life, to struggle against that which obstructs its preservation and development taken as norms.... the fact that a living man reacts to a lesion, infection, functional anarchy by means of a disease expresses the fundamental fact that life is not indifferent to the conditions in which it is possible, that life is polarity and thereby even an unconscious positing of value; in short, life is in fact a normative activity [[14], pps. 338-339].

Since we have mentioned *feeling*, we would like to conclude by indicating its importance for any philosophy of life. Although it is difficult to pinpoint the precise level, at some level of life the organism's relationship with the world becomes a relationship of *feeling*: many organisms are *sensitive to *elements in their environments. Again this applies to individual cells as well as to conglomerates of cells and whole organisms. Sensitivity is the first glimmering of *subjectivity *in organisms, if we may apply the word "subjectivity" to even the most primitive and elemental kinds of feeling. And as we move up the living kingdom to more and more complex organisms, sensitivity too becomes more complex; and at a certain point we can speak of organisms *perceiving *items composing the environment. It would, of course, be difficult to mark the progressive difference between an elemental *sensitivity *to the outside and an actual *perception *of it, for any form of felt sensitivity may already count as an *experience*, at least of a very basic sort. Our point here is, however, that the first glimmerings of subjectivity arise relatively early in the phylogenetic scale. And once subjectivity appears, it grows in complexity, refinement, and acuity. "Mind," then, is certainly not the exclusive privilege of human beings. It is not even the exclusive possession of the higher animals. Mental life begins where sensitivity to the outside is felt [8,15].

This birth of subjectivity marks another aspect of the selfhood of living beings. For as subjectivity grows and becomes more complex, the organism is able to sense its environment across spatial distances and to feel a desire for things across time. If we add to this subjectivity the movement of the organism's body, then the living being can move across the spatial distances and purse objects as long as desires for them are felt. With growing experience and motility, then, livings beings confront a world that grows in its spatial extent and its temporal duration. Mind renders organic world-relatedness richer and more encompassing, even if this larger exposure to the outside also expands the realm from which threats to life can emerge [15].

Drawing extensively on the philosophy of life of Hans Jonas, we have traced the development of living forms from their most basic constituents up to the more complex forms in which mental life, with an increasing span of feeling, desire, and perception, connect the living organism with an increasing variety of things and events, things and events which both nurture and threaten it. In subsequent essays we shall provide a more ample conception of how human life enriches and changes this development through the construction and assimilation of culture. However, we hope that the present sketch furnishes the fundamentals for a unified understanding of life that avoids mind-body dualism. Such an understanding, we maintain, is essential for a psychosomatic medicine that can encompass all the factors that affect health and illness.

## Competing interests

No authors have affiliations or financial involvement with any organization or entity with a direct financial interest in the subject matter or materials discussed in the manuscript.

The authors declare that they have no competing interests.

## Authors' contributions

Both authors contributed equally to this work. Both authors read and approved the final manuscript.

## Authors' information

Michael Alan Schwartz, M.D. and Osborne P. Wiggins, Ph.D have been collaborating authors for almost twenty five years, since their initial essay, "Science, Humanism and the Nature of Medical Practice: A Phenomenological View," was published in Perspectives in Biology and Medicine 28:331-361, 1985. (The essay was also reprinted in Odegaard CE: Dear Doctor: A Personal Letter to a Physician The Henry J. Kaiser Family Foundation, Menlo Park, California, pps. 117-156, 1987). In 1989, Dr. Schwartz and Professor Wiggins were among five co-founders of the Association for the Advancement of Philosophy and Psychiatry; and in 1998, they shared the Dr. Margrit Egnér-Stiftung Prize given at the University of Zurich for "contributing with their work to a more human world in which the human being with its mental needs stands in the center." Michael Schwartz, who practices psychiatry in Austin, Texas, is a Clinical Professor of Psychiatry at the University of Hawaii, and a Founding Editor of "Philosophy, Ethics, and Humanities in Medicine (PEHM)". Osborne Wiggins is Professor of Philosophy at the University of Louisville and a member of the Associate Faculty at Louisville's Institute for Bioethics, Health Policy, and Law.
